# Cannabidiol Administration Prevents Hypoxia-Ischemia-Induced Hypomyelination in Newborn Rats

**DOI:** 10.3389/fphar.2019.01131

**Published:** 2019-09-26

**Authors:** María Ceprián, Carlos Vargas, Laura García-Toscano, Federica Penna, Laura Jiménez-Sánchez, Svein Achicallende, Izaskun Elezgarai, Pedro Grandes, William Hind, M. Ruth Pazos, José Martínez-Orgado

**Affiliations:** ^1^Department of Experimental Medicine, Health Research Institute Puerta de Hierro Majadahonda, Madrid, Spain; ^2^Departamento de Bioquímica y Biología Molecular, Facultad de Medicina, Instituto Universitario de Investigación en Neuroquímica, Universidad Complutense, Madrid, Spain; ^3^Division of Neonatology, Hospital Clínico San Carlos - IdISSC, Madrid, Spain; ^4^CIBER de Enfermedades Neurodegenerativas (CIBERNED), Madrid, Spain; ^5^Instituto Ramón y Cajal de Investigación Sanitaria (IRYCIS), Madrid, Spain; ^6^Department of DBSV, Laboratory of Neuropsychopharmacology, University of Insubria, Varese, Italy; ^7^School of Medicine and Nursery, Universidad del País Vasco, Bilbao, Spain; ^8^GW Research Ltd, Cambridge, United Kingdom; ^9^Laboratorio de Apoyo a la Investigación, Hospital Universitario Fundación Alcorcón, Madrid, Spain

**Keywords:** hypoxia-ischemia, myelin, cannabidiol, newborn, rat

## Abstract

Neonatal hypoxia-ischemia (HI) is a risk factor for myelination disturbances, a key factor for cerebral palsy. Cannabidiol (CBD) protects neurons and glial cells after HI insult in newborn animals. We hereby aimed to study CBD’s effects on long-lasting HI-induced myelination deficits in newborn rats. Thus, P7 Wistar rats received s.c. vehicle (HV) or cannabidiol (HC) after HI brain damage (left carotid artery electrocoagulation plus 10% O_2_ for 112 min). Controls were non-HI pups. At P37, neurobehavioral tests were performed and immunohistochemistry [quantifying mature oligodendrocyte (mOL) populations and myelin basic protein (MBP) density] and electron microscopy (determining axon number, size, and myelin thickness) studies were conducted in cortex (CX) and white matter (WM). Expression of brain-derived neurotrophic factor (BDNF) and glial-derived neurotrophic factor (GDNF) were analyzed by western blot at P14. HI reduced mOL or MBP in CX but not in WM. In both CX and WM, axon density and myelin thickness were reduced. MBP impairment correlated with functional deficits. CBD administration resulted in normal function associated with normal mOL and MBP, as well as normal axon density and myelin thickness in all areas. CBD’s effects were not associated with increased BDNF or GDNF expression. In conclusion, HI injury in newborn rats resulted in long-lasting myelination disturbance, associated with functional impairment. CBD treatment preserved function and myelination, likely as a part of a general neuroprotective effect.

## Introduction

Neonatal hypoxia-ischemia (HI) is a major cause of neonatal encephalopathy (Volpe 2012; Lee et al., 2013). Newborn hypoxic-ischemic encephalopathy (NHIE) has an incidence of 2 to 6 per 1000 live births (Kurinczuk et al., 2010; Lehtonen et al., 2017). About 25% of affected newborns develop long-lasting disabilities, such as cerebral palsy, cognitive impairments, or epilepsy with deleterious consequences for the child and family well-being (de Vries and Jongmans, 2010; Martinez-Biarge et al., 2011; Lee et al., 2013; Adhikari and Rao, 2016). The complex pathophysiology of HI brain damage includes the so-called deadly triad oxidative stress, excitotoxicity, and severe inflammatory response (Fatemi Fatemi et al., 2009; Hagberg et al., 2015), to which late oligodendrocyte progenitors (preOL) are particularly sensitive because of the combination of high metabolic demand, undeveloped antioxidant system, specific subcellular distribution of glutamatergic receptors [including *N*-methyl--aspartate (NMDA) and alpha-3-amino-hydroxy-5-methyl-4-isoxazole propionic acid (AMPA)] and particular pro-apoptotic effects of cytokines, such as TNFα or IFNγ (Back et al., 2002a; Buntinx et al., 2004; Fragoso et al., 2004; Follett et al., 2004; Salter and Fern 2005; Kim et al., 2011; Li et al., 2013; Janowska and Sypecka, 2018).

Thus, preOL degeneration is enhanced in the brain, both in animal models and human newborns after HI damage (Segovia et al., 2008; Back et al., 2002a), which eventually leads to hypomyelination (Janowska and Sypecka, 2018). In the core of the lesion, axonopathy has been observed to overlap with hypomyelination in microscopic necrosis region (Buser et al., 2012; Back et al., 2002a). Furthermore, this damage in white and grey matter has been correlated with a poorer pathology outcome, such as motor impairment, delay in language, and behavioral problems (de Vries and Jongmans, 2010; Martinez-Biarge et al., 2011).

Cannabidiol (CBD) is the main non-euphoric component of *Cannabis sativa* and is well-known as a potent anti-oxidant and anti-inflammatory compound (Mechoulam et al., 2002; Mukhopadhyay et al., 2011), properties involved, together with modulation of excitotoxicity, in the neuroprotective effects demonstrated for CBD in different animal models of NHIE (Alvarez et al., 2008; Lafuente et al., 2011; Pazos et al., 2012; Pazos et al., 2013; Lafuente et al., 2016). CBD treatment has myelin protective effects in different demyelinating disease models. CBD prevents oligodendrocyte progenitor (OPC) death by both inflammatory and oxidative stress *in vitro* (Mecha et al., 2012). In animal models of multiple sclerosis, CBD by itself or co-administrated with other cannabinoids improves the neurobehavioral outcome, diminishing demyelination severity and axonal damage by reducing microglia activation and proinflammatory cytokines release (Feliú et al., 2015; Giacoppo et al., 2015; Rahimi et al., 2015). However, no assessment of CBD’s effects on myelination and oligodendrocyte protection was reported in those studies.

The aim of the present work is to determine if CBD may prevent HI-induced hypomyelination in immature brain. Mature oligodendrocyte population and myelin density are characterized in white and grey matter where specific areas have been analyzed. Axonopathy and myelin abnormalities are studied by electron microscopy to characterize long-term consequences of impaired oligodendrocyte maturation.

## Materials and Methods

### HI Brain Damage Induction

All the animal experimental procedures were approved by the Ethical Committee for Animal Welfare of the Hospital Universitario Puerta de Hierro Majadahonda, and met the European and Spanish regulation (2010/63/EU and RD 1201/2005). HI brain damage protocol was based on that previously reported (Pazos et al., 2012). In brief, 7- to 10-day-old Wistar rats (P7–P10) were anesthetized with sevoflurane (5% induction, 1% maintenance) and the left common carotid artery was exposed and electrocoagulated. After a 3-h recovery period, pups were placed into 500ml jars in a 37°C water bath and exposed to hypoxia (10% O_2_) for 112 min. Similar surgical procedures were performed for control animals (SHM, n = 16), but without carotid electrocoagulation and the following hypoxia. Ten minutes after the end of hypoxia, HI pups were randomly treated with s.c. injection of pure CBD (HC, n = 29) or vehicle (HV, n = 27). CBD (GW Research Ltd, Cambridge, UK) was obtained from a 5-mg/ml formulation of CBD in ethanol/solutol/saline (2:1:17) and further diluted in saline to inject 1 mg/kg in 0.1 ml final volume Then, rats were returned to the dam and weaned at P21 days.

### Neurobehavioral Studies (NB)

Thirty days after the Hi insult, two different functional tests were performed, following the protocol previously described (Pazos et al., 2012). Briefly, lateral bias of sensorimotor deficits was assessed using the cylinder rearing test (CRT). Each rat was placed in a methacrylate transparent cylinder 20 cm in diameter and 30 cm in height. The relative proportion of left (ipsilateral) forepaw contacts was calculated as: [(left − right)/(left + right + both)] × 100.

Then, non-spatial working memory was assessed using the novel object recognition (NOR) test. The rat was allowed to explore a box that contained two identical objects and then returned to the box where one of the original objects was replaced by a new one. The time spent for the exploration of the familiar (Tf) and the novel object (Tn) was recorded separately, and a discrimination index, [(Tn − Tf)/(Tn + Tf)] × 100, was calculated.

### Immunohistochemical Analyses

After the NB studies were completed, rats were sacrificed under deep anesthesia (i.p. injection of diazepam/ketamine) and transcardially perfused with saline and 4% paraformaldehyde. Brains were harvested and embedded in paraffin to obtain coronal sections (4 µm thick) corresponding to plate 17 of the Paxinos and Watson Atlas (Paxinos and Watson, 1996). Mature oligodendrocyte (mOL) populations were studied in the ipsilateral cortex (CX) (parieto-occipital cortex) and white matter (WM) (external capsula of the corpus callosum) using the immunohistochemistry protocol described elsewhere (Pazos et al., 2012). Briefly, tissue sections were deparaffinized and, after an antigen retrieval procedure, were incubated overnight at room temperature with GST-p (1:100; Abcam, UK) or MBP antibodies (1:800; Abcam, UK). After extensive washes, tissues were incubated with the corresponding Alexa-Fluor conjugated secondary antibody (1:200; Life Technologies, Spain) for 2 h at 37°C. Finally, TO-PRO (1:500; Life Technologies, Spain) was used for nucleus staining. A Leica TCS SP5 confocal microscope system (Leica, Wetzlar, Germany) was used for slide observation and photography. Three microphotographs were obtained for each area of interest for at least eight animals per group. Cell density analyses were performed by at least two blinded examiners using the ImageJ 1.43u software (NIH, Bethesda, USA). MBP signal intensity was determined with the LEICA LASF Software (Leica Microsystems, Germany) and expressed as ratio of ipsilateral MBP intensity compared to total intensity(Liu et al., 2002).

### Electron Microscopy Studies

Electron microscopy (EM) studies were performed at P30 as described elsewhere (Ford et al., 2015). In brief, rats were deeply anesthetized and transcardially perfused with phosphate-buffered saline followed by the fixative solution (4% formaldehyde, 0.2% picric acid, and 0.1% glutaraldehyde in 0.1 M phosphate buffer, pH 7.4). Afterward, brains were removed from the skull and postfixed in the fixative solution for approximately 1 week at 4°C. Brain coronal sections were cut in a vibratome (50 μm thick) and immersed in a 1% osmium tetroxide solution for 1 h at 4°C. Then, sections were dehydrated in graded alcohol series to propylene oxide and plastic-embedded in Epon resin 812. Ultrathin sections of 60 nm were collected on mesh nickel grids, stained with 2.5% lead citrate for 20 min, and examined in a Philips EM208S electron microscope. Tissue preparations for three animals per treatment were photographed by using a digital camera coupled to the electron microscope. Two main areas were analyzed, external capsule and cerebral cortex, and all the ultramicrographs were analyzed using ImageJ software (NIH; RRID: SCR_003070).

To calculate the number of axons, 10 EM images per animal were taken at 5,600× magnification. The number of cross-sectional views of myelinated axons were counted per area (308 μm^2^) to normalize the values. All the EM micrographs used to analyze myelin sheath thickness and axon perimeter were taken at 11,000× magnification. Myelin sheath thickness was analyzed in 10 axons per animal, and it was measured in four different points of the same axon. To estimate g-ratios, the measured inner perimeter (axon) was divided by the measured outer perimeter (myelin). 150 axons per treatment were analyzed in the external capsule, whereas in the cortex an average of 100 axons were analyzed.

### Western Blot

Brain-derived neurotrophic factor (BDNF) and glial cell-derived neurotrophic factor (GDNF) expression was studied one week after the HI insult (Ikeda et al., 2002; Jantzie and Todd 2010). Rats were sacrificed by carbon dioxide followed by a rapid decapitation. Brains were immediately harvested, snap frozen in isopentane, and stored at -80°C to carry out biochemical studies using the protocol described elsewhere (Pazos et al., 2012; Pazos et al., 2013). 20 µg of protein from each frozen tissue sample was denatured and separated by sodium dodecyl-sulfate-polyacrylamide gel electrophoresis (SDS-PAGE). Proteins were transferred onto a PVDF membrane (GE Healthcare; UK), and remaining binding sites were blocked by one h incubation in TBS-Tween (TBST) containing 4% nonfat dried milk at room temperature. Subsequently, blots were incubated with α-BDNF (1:300; Santa Cruz, USA) or α-GDNF (1:100; Santa Cruz, USA). Membranes were finally incubated with the corresponding HRP-labeled secondary antibodies (1:5000; GE Healthcare, UK) for 2 h at room temperature. Finally, the peroxidase reaction was developed with an Enhance Chemiluminescence (ECL) Kit (GE Healthcare; Buckinghamshire, UK) and Gel-Doc station with Quantity One 4.5.1 (basic) analysis software (Bio-Rad; Hercules, USA) was used for detection. Stain-free technology was used to normalize sample loading (BioRad, Spain) and obtained images of both target protein and loaded protein were analyzed with ImageLab software (version 6.0.0, Bio-Rad; Hercules, USA) to determine the intensity per mm^2^ of each band and lane, respectively.

### Statistical Analyses

Statistical analysis was done using the StatPlus:mac Pro v6.1.25 software (AnalystSoft Inc., Walnut, CA). Normality of data was tested using the Kolmogorov-Smirnov test. Since no evidence against normality was detected, comparisons between groups were made using one-way ANOVA with Bonferroni *post hoc* analysis for multiple comparisons whereas Spearman test was used to analyze the correlation between myelin intensity and neurobehavioral studies. Results are expressed as mean ± SEM. *p* < 0.05 was considered statistically significant.

## Results

Experimental mortality accounted for 15.24% of animals: 5.71% died during the surgical procedure, 4.76% during hypoxia, and 4.76% over the following 7 days.

### WM Findings

**Figure 1:**
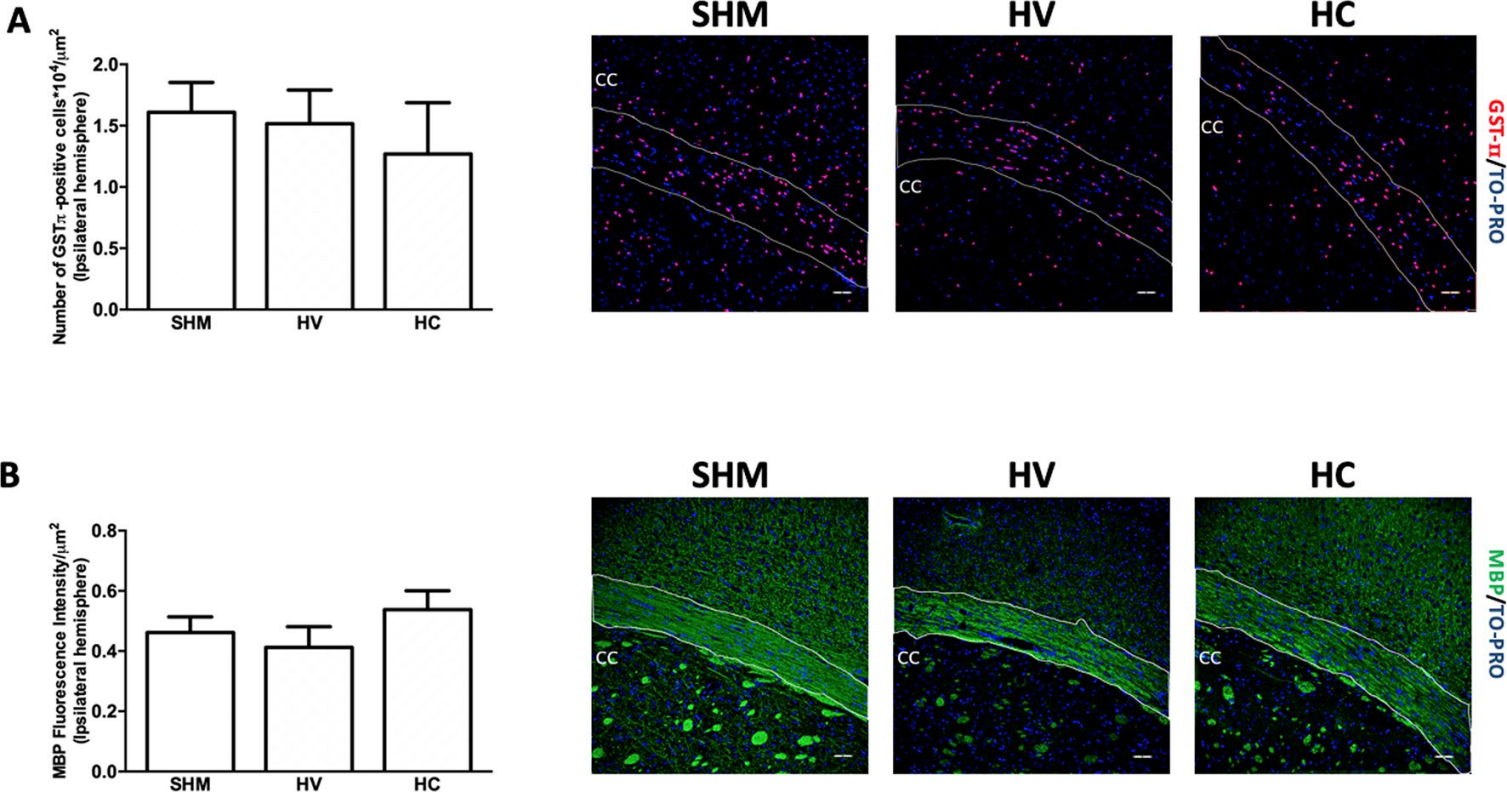
Quantification of mature oligodendrocyte population and myelin staining in white matter. Representative microphotographs and graphical representation of immunohistochemical studies performed in the external capsula of the corpus callosum 30 days after a hypoxic-ischemic (HI) insult induced in P7–10 Wistar rats then receiving vehicle (HV) or cannabidiol (HC), or a similar period in control rats (SHM). **(A)** GST-π (mature oligodendrocyte) and **(B)** myelin basic protein (MBP) staining. Bars represent mean ± SEM. Scale bar: 50 μm.

**Figure 2:**
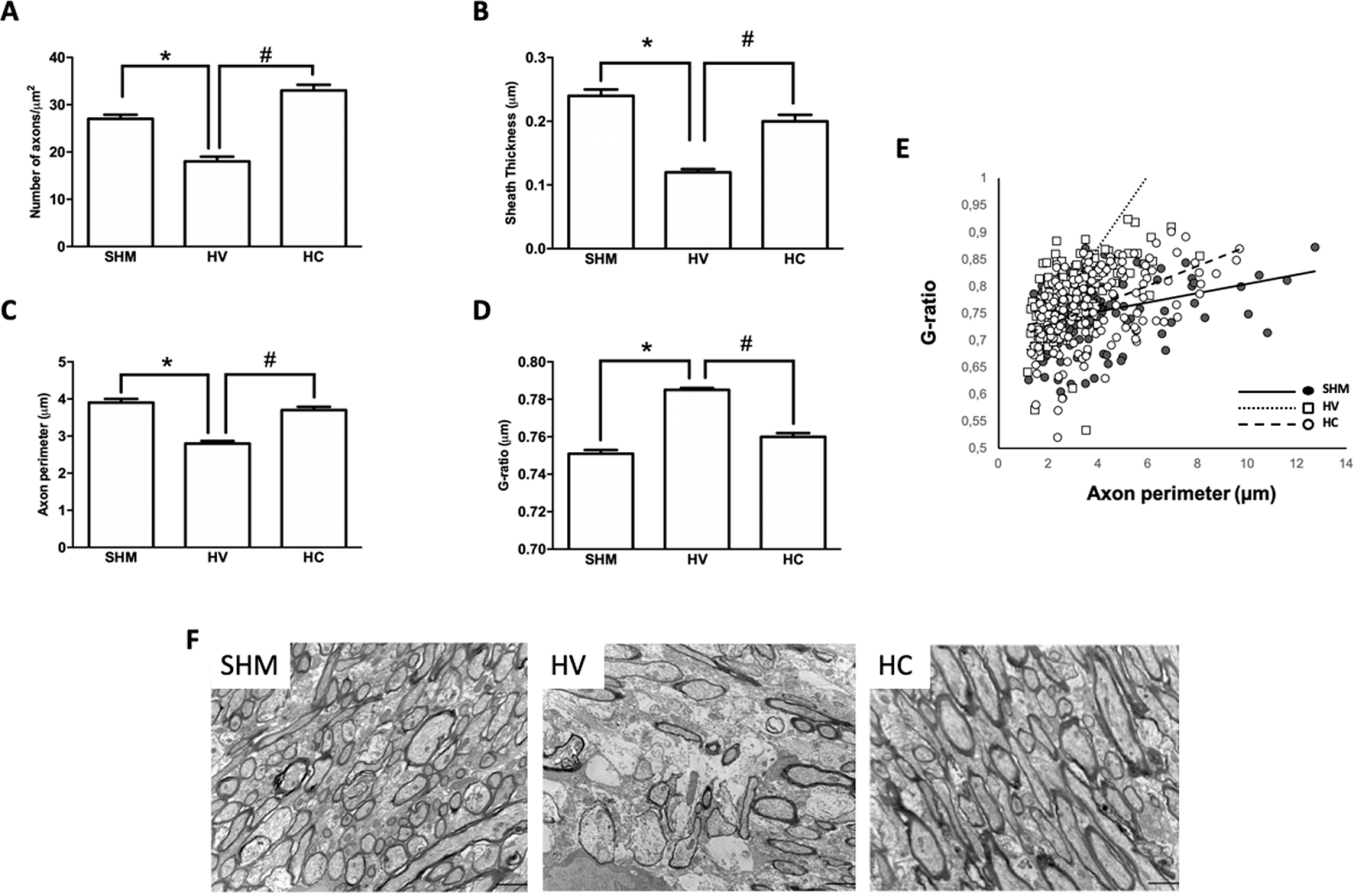
Myelin study in white matter by electron microscopy. Electron microscopy studies performed in the external capsula of the corpus callosum 30 days after a hypoxic-ischemic (HI) insult induced in P7–10 Wistar rats then receiving vehicle (HV) or cannabidiol (HC), or a similar period in control rats (SHM). **(A)** Number of axons **(B)** myelin sheath thickness **(C)** Axon perimeter **(D)** g-ratio **(E)** Dots and trend lines representing the relationship between axon size and g-ratio values **(F)** Representative micrographs. Bars represent mean ± SEM. Scale bar: 10 µm. *p < 0.05 vs SHM; ^#^*p* < 0.05 vs HV, by ANOVA.

Density of GSTπ-positive cells (**Figure 1A**) and MBP intensity signal (**Figure 1B**) were similar in HV or HC and SHM. However, EM studies (**Figure 2F**) revealed that despite the normal MBP intensity signal, there was in WM a decrease in the number of axons in HV as compared to SHM 1 month after HI (**Figure 2A**). In addition, HV axons presented a thinner myelin sheath than those from SHM (**Figure 2B**). Since axon perimeter was also smaller in HV than in SHM animals (**Figure 2C**), myelin sheath thickness was normalized using the g-ratio. G-ratio was greater in HV than in SHM, confirming that myelin sheath was actually thinner in HV than in SHM animals (**Figure 2D**). EM studies revealed a beneficial effect of CBD, so that the number of myelinated axons as well as myelin thickness in absolute values or as g-ratio, were similar in HC and SHM animals (**Figures 2A−D**). In HV animals, smaller axons showed a trend to present higher g-ratio values (**Figure 2E**) whereas in HC animals the relationship between axon size and g-ratio values was similar to SHM (**Figure 2E**). Axon size correlated with g-ratio value in all groups (R = 0.32, t = 4.0; R = 0.59, t = 8.9; and R = 0.48, t = 7.1, for SHM, HV, and HC, respectively, all *p* < 0.05)

### Cortex Findings

**Figure 3:**
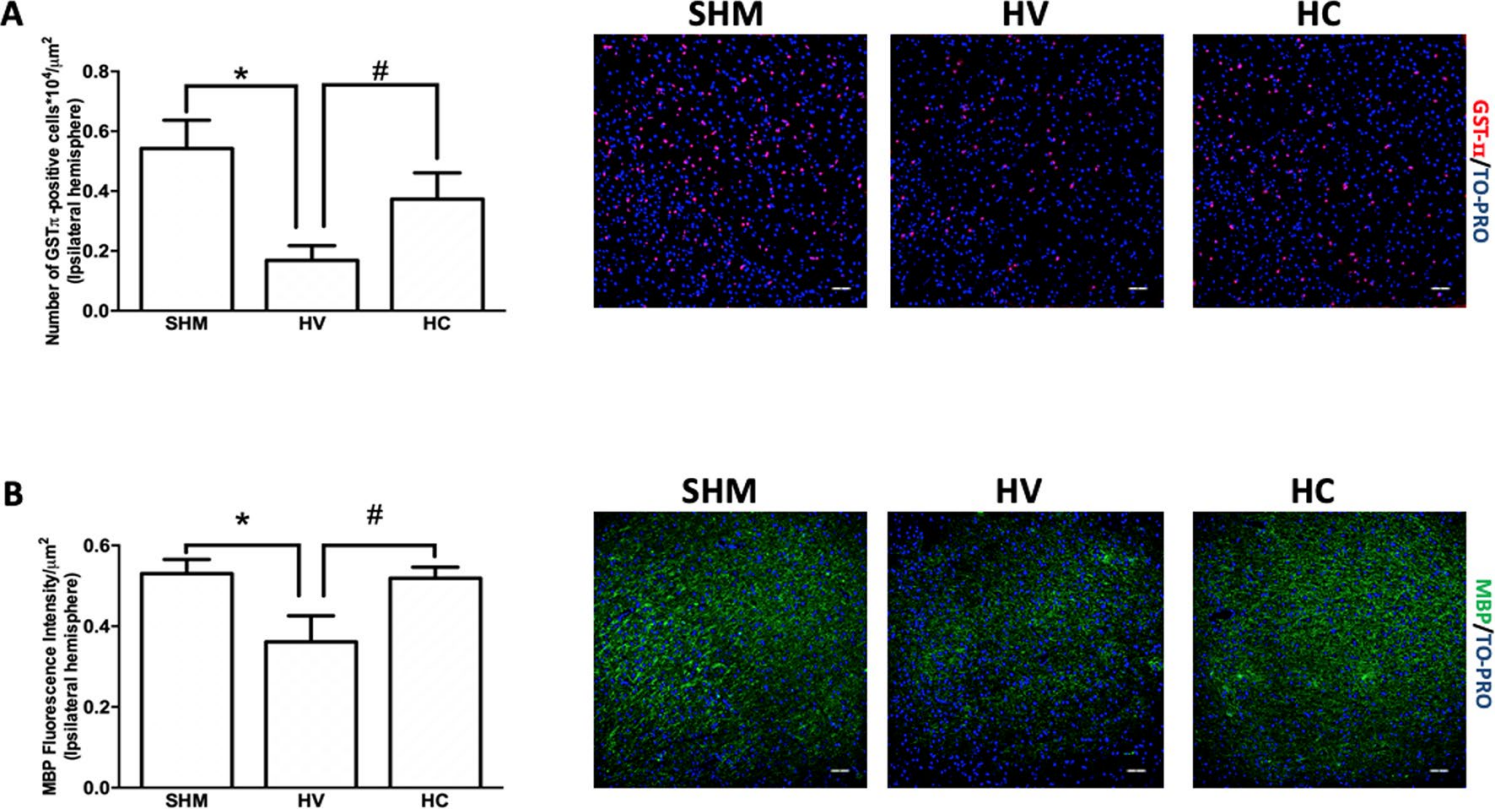
Quantification of mature oligodendrocyte population and myelin staining in cerebral cortex. Representative microphotographs and graphical representation of immunohistochemical studies performed in the parieto-occipital cortex 30 days after a hypoxic-ischemic (HI) insult induced in P7–10 Wistar rats then receiving vehicle (HV) or cannabidiol (HC), or a similar period in control rats (SHM). **(A)** GST-π (mature oligodendrocyte) and **(B)** myelin basic protein (MBP) staining. Bars represent mean ± SEM. Scale bar: 50 μm. *p < 0.05 vs SHM; ^#^ *p* < 0.05 vs HV), by ANOVA.

In this area, the density of GSTπ-positive cells was lower in HV than in SHM (**Figure 3A**), which is in contrast to that observed in WM. In addition, MBP intensity signal was reduced in HV as compared to SHM (**Figure 3B**). CBD treatment prevented the HI-induced decrease in GSTπ-positive cell number so that the number of GSTπ-positive cell was similar in HC and SHM (**Figure 3A**). Similarly, CBD treatment prevented the HI-induced decrease of myelin signal so that MBP intensity signal was similar in HC and SHM (**Figure 3B**).

**Figure 4:**
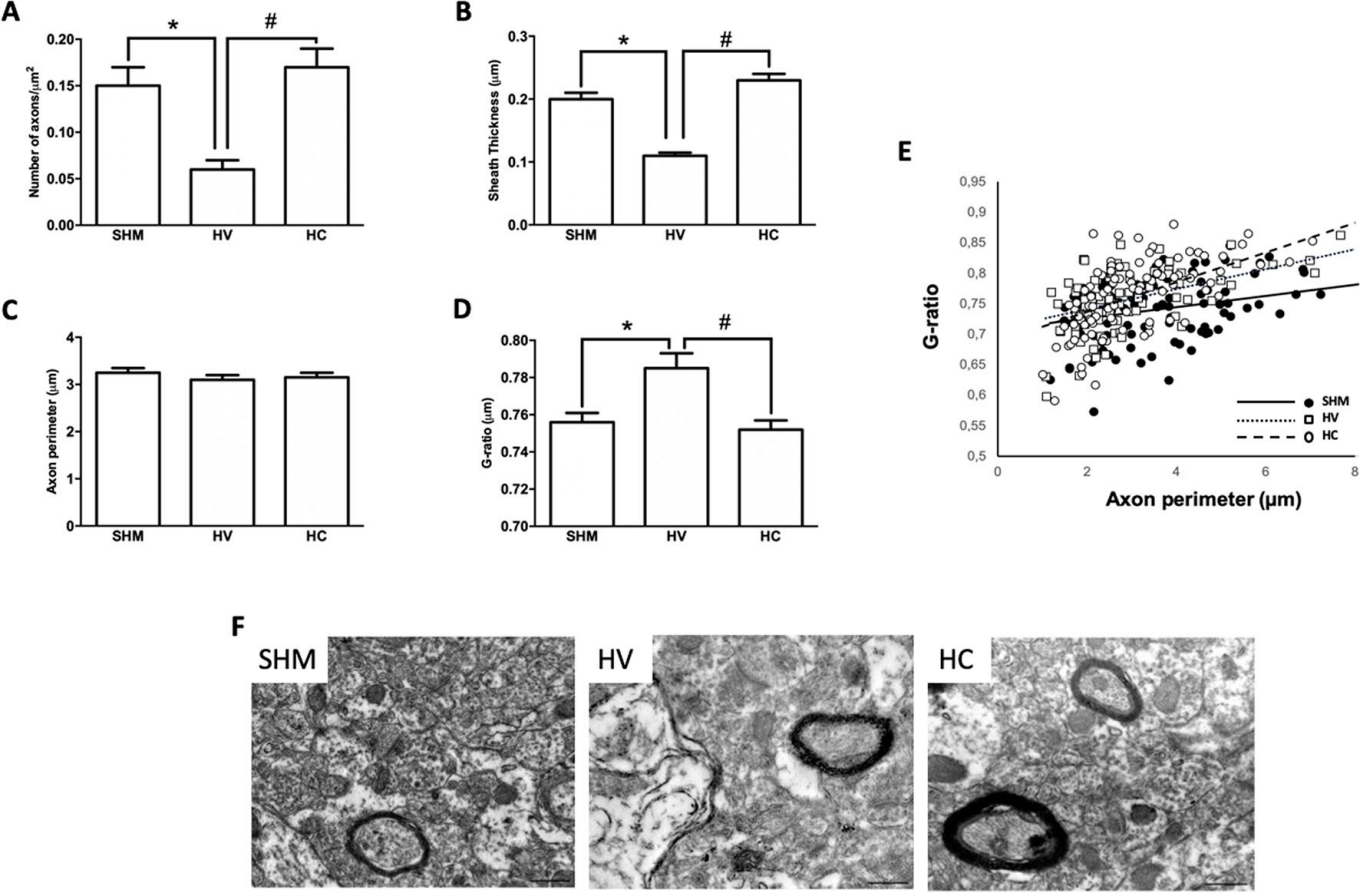
Myelin study in cerebral cortex by electron microscopy Electron microscopy studies performed in the parieto-occipital cortex 30 days after a hypoxic-ischemic (HI) insult induced in P7–10 Wistar rats then receiving vehicle (HV) or cannabidiol (HC), or a similar period in control rats (SHM). **(A)** Number of axons **(B)** myelin sheath thickness **(C)** Axon perimeter **(D)** g-ratio **(E)** Dots and trend lines representing the relationship between axon size and g-ratio values **(F)** Representative micrographs. Bars represent mean ± SEM. Scale bar: 10 µm. *p < 0.05 vs SHM; #*p* < 0.05 vs HV), by ANOVA.

As in WM, EM studies (**Figure 4F**) in the CX showed a decrease in the number of axons in HV as compared to SHM (**Figure 4A**). Myelinated axons in CX showed a thinner myelin sheath in absolute terms (**Figure 4B**) and after normalization to g-ratio (**Figure 4D**). CBD administration after the HI insult prevented these changes from occurring in the CX, with myelinated axon number as well as myelin thickness in absolute values or as G-ratio similar in HC and SHM animals (**Figures 4A−D**). Relationship between axon size and g-ratio values was similar in all groups (**Figure 4E**). Axon size correlated with g-ratio value in all groups (R = 0.32, t = 3.5; R = 0.54, t = 5.6; and R = 0.58, t = 7.2, for SHM, HV, and HC, respectively, all *p* < 0.05)

### Functional Studies

HI led to impaired NB performance both in motor (CRT: -1.3 ± 4.8 vs 1.5 ± 4.7% for SHM and HV, respectively, *p* < 0.05) and cognitive tests (NOR: 66.0 ± 4.0 vs. 46.5 ± 6.2% for SHM and HV, respectively, *p* < 0.05). CBD restored NB performance (CRT: -0.72 ± 5.3%, *p* < 0.05 vs. HV; NOR: 61.6 ± 5.2%; both *p* < 0.05 vs. HV and NS vs. SHM).

**Figure 5:**
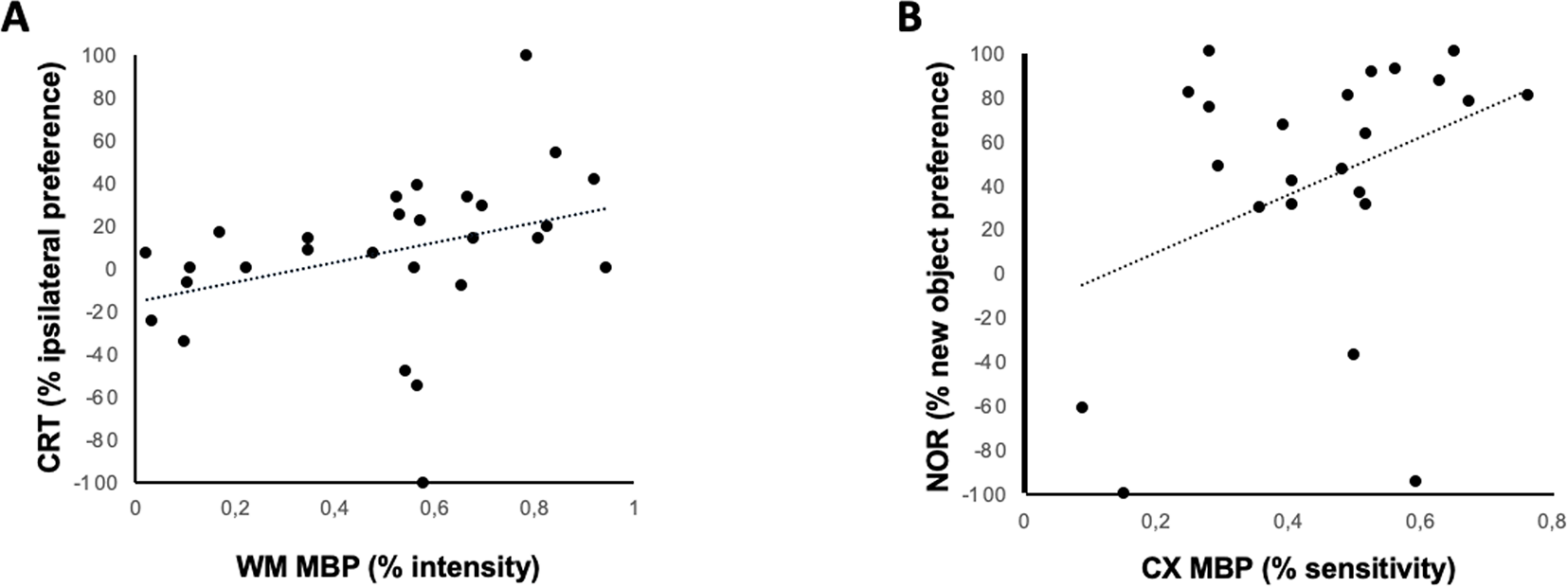
Relationship between immunohistochemical and neurobehavioral studies. Linear correlation between MBP staining intensity and different neurobehavioral test performed 30 days after a hypoxic-ischemic insult induced in P7–10 Wistar rats or a similar period in control rats. **(A)** Correlation between hemiparesis as assessed by the cylinder rearing test (CRT) and MBP intensity in White Matter (corpus callosum, CC); Spearman’s correlation: R = 0.396, t = 2.58, *p* = 0.013. **(B)** Correlation between memory impairment as assessed by novel object recognition test (NOR) and MBP intensity in cortex; Spearman’s correlation: *R* = 0.443, t = 2.57, *p* = 0.015).

A significant relationship was found 1 month after HI between MBP signal intensity in EC and the severity of hemiparesis, as assessed by CRT (R = 0.39, t = 2.6, *p* < 0.05) (**Figure 5A**), and between MBP signal intensity in CX and the severity of memory impairment, as assessed by NOR (R = 0.44, t = 2.6, *p* < 0.05) (**Figure 5B**).

### Biochemical Studies

**Figure 6:**
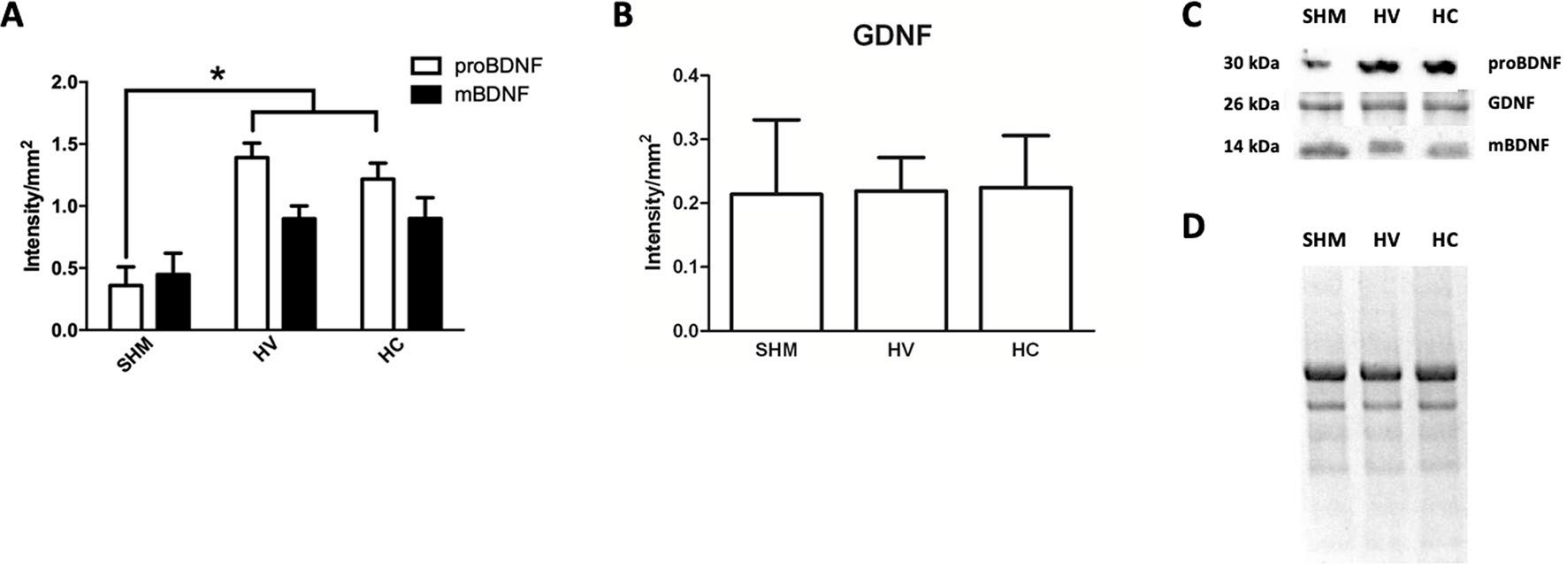
Determination of neuroproliferative factor expression. Western blot studies performed in the parieto-occipital cortex seven days after a hypoxic-ischemic (HI) insult induced in P7–10 Wistar rats then receiving vehicle (HV) or cannabidiol (HC), or a similar period in control rats (SHM). **(A)** Quantitative determination of the precursor (proBDNF) and mature (mBDNF) isoforms of brain-derived neurotrophic factor (BDNF) expression **(B)** Quantitative determination of glia-derived neurotrophic factor (GDNF) expression. Bars represent mean ± SEM. **(C)** Representative Western blot of proBDNF and mBDNF, GDNF. **(D)** Representative stain-free total protein image. *p < 0.05 vs SHM, by ANOVA.

In Western Blot studies (**Figure 6C-D**) BDNF in its precursor form (proBDNF) was increased one week after the HI insult (**Figure 6A**). By contrast, no differences were observed between HV and SHAM when comparing the expression of its mature form (mBDNF) (**Figure 6A**). GDNF protein expression was not affected by HI seven days after HI (**Figure 6B**). CBD administration after HI had no effect on BDNF or GDNF protein expression so that levels in HC were similar to HV (**Figures 6A−C**).

## Discussion

Results from the present work have a double relevance. First, we hereby confirmed that acute HI insult led to the impairment of myelination in rat brains at a developmental stage comparable to that of term human infants. This is noteworthy since most studies on myelination impairment have focused on WM injury in models of very preterm or late preterm newborns (Back et al., 2002a; Back et al., 2002b; Segovia et al., 2008; Back 2014; Back and Rosenberg 2014). In addition, we present for the first time a relationship between mOL damage and long-term functional impairment, suggesting a role for acute HI-induced hypomyelination on long-lasting sequelae after acute brain HI insult in newborns. Second, we demonstrated that CBD has beneficial effects in neonatal brain including OL protection, which complements the previously demonstrated protective effects of CBD on neurons and astrocytes (Schiavon et al., 2014; Lafuente et al., 2011; Ceprián et al., 2017). Beneficial effects of CBD on myelination, in addition, would open exciting perspectives regarding a possible role for CBD in different pediatric conditions related with myelin damage.

HI insult differentially affected OL in WM and CX. One month after the HI insult, the density of mOL cells in CX was reduced in HI compared to control rats. The reduction in the number of mOL resulted in reduced myelin production as reflected by the low MBP immunostaining signal found in CX in HI as compared to SHM rats. By contrast, in WM the density of mOL was similar in HI and SHM rats. Accordingly, quantitative myelin production was preserved, as reflected by the similar MBP immunostaining signal found in WM in HI and SHM rats. Such differences between WM and CX could be explained by the different maturational stage of OL in WM and CX at term (Segovia et al., 2008; Buser et al., 2010; Tomassy and Fossati, 2014). Maturation from preOL to immature OL (iOL) in WM starts by P2–P3 (Back et al., 2002a; Back et al., 2002b), whereas in CX preOL maturation happens by P7–P10 (Segovia et al., 2008; Tomassy and Fossati, 2014). Thus, when the HI insult was induced in our experiments (at P7–10) most of the maturational process from preOL to mOL was already accomplished in WM. Since mOL are far more resistant to the HI insult than preOL, the HI insult did not result in a decreased number of mOL, in agreement with previous reports (Fragoso et al., 2004; Alix 2006; Back et al., 2007; Volpe, 2011; Back and Rosenberg, 2014). In CX, by contrast, the HI insult occurred precisely when the maturational process from preOL to iOL is taken place. Due to the particular sensitivity of late preOL to HI insults, this likely resulted in impaired maturation form preOL to iOL, leading to a reduced number of mOL in CX, as reported (Back et al., 2002a; Xiong et al., 2013).

We further investigated the effects of acute HI on myelin production by studying axons and myelin sheath using EM. In agreement with our findings in IHC studies, in CX there was a remarkable reduction of myelinated axon number as well as a reduction of axonal myelin sheath thickness, as shown by a greater g-ratio in HI than in SHM rats. Interestingly, similar results were obtained in WM, including a thinner myelin sheath in axons in HI than in SHM rats as reflected by the greater g-ratio. Such a reduction of myelin sheath thickness was subtle enough to not significantly reduce gross myelin production as quantified by MBP immunostaining, but suggests that, even though the number of mOL was not reduced in WM by HI, myelination was impaired after HI. Myelination is still in progress after birth with OL extending their processes to axons and developing the myelin sheath (Back et al., 2002b). This process is very sensitive to excitotoxicity and inflammation (Miron et al., 2013; Jantzie et al., 2015), which are still present in the immature brain several days after the HI insult (Hedtjarn et al., 2002; Winerdal et al., 2012). In WM, the more sensitive axons to those insults are those preparing to myelinate, which are those with a smaller diameter (Alix et al., 2012). HI-induced damage of those axons interferes with the appropriate cross-talk between axon and OL resulting in impaired myelination even in absence of gross reduction of OL cell density (Alix et al., 2012). This in agreement with the results of our study, in which HV animals showed in WM a trend to present higher g-ratios in the smaller axons as compared to SHM despite a similar density of OL cells. By contrast, in CX impaired myelination resulted from the direct damage of mOL, as showed by the reduction of mOL cell density in HV with no differences in the axon size-g-ratio relationship.

Another remarkable finding of our work is that we present, for the first time, a direct relationship between myelination impairment and functional sequelae after an acute HI insult in rats with a developmental stage comparable to term infants. Conceivably, myelin density at WM correlated with hemiparesis severity, since the WM constituted the path for axons coming from cortex motor neurons to the medulla (Brus-Ramer et al., 2009). Interestingly, myelin density at parietal CX correlated with working memory impairment, as assessed by the NOR test. NOR test is impaired in rats suffering from HI insult at the neonatal period, this finding being attributed to hippocampus damage (Pazos et al., 2012). However, parietal and other different cortical areas are determinant in memory circuitry too by influencing perirrhinal and entorrhinal cortex actions on hippocampus (Dere et al., 2007).

It is noteworthy that administration of CBD prevented the HI-induced disturbance of myelination. Thus, HI rats receiving CBD did not show the quantitative reduction of myelin production observed in CX form HV rats 1 month after HI. More importantly, CBD administration after the HI insult at P7–10 resulted in a normal number of myelinated axons and normal axonal myelin sheath thickness at P37 in both WM and CX. In WM, CBD treatment did not modify mOL cell density but resulted in a similar pattern and relationship between axon size and g-ratio than that of SHM, suggesting that in WM, rather than preserving the density of mOL cells CBD was protecting the axons preparing to myelinate thus preserving an appropriate myelination. In CX, CBD treatment did restore mOL cell density, suggesting that in this area protection of OL cell maturation was one of the effects of CBD treatment. There are no reports on the effect of CBD on myelination after acute brain insults, although there is evidence for CBD reducing demyelination and axonal loss in spinal cord in inflammation-mediated models of multiple sclerosis in mice (Giacoppo et al., 2015; Feliú et al., 2015; Rahimi et al., 2015). *In vitro*, CBD reduces preOL apoptosis by reducing oxidative stress because of its antioxidant properties and reducing inflammation damage by modulating endoplasmic reticulum stress (Mecha et al., 2012). We have previously reported in this model that post-HI administration of CBD results in a reduction of both glutamate and TNFα concentration seven days after the HI insult (Pazos et al., 2012). Furthermore, we have observed that CBD administration modulated oxidative stress over the days following HI, so that at P14 protein nitrosylation in HC rats was similar to control and lower than in HV rat (Téllez et al., unpublished data). OL are particularly sensitivity to oxidative stress because of their undeveloped antioxidant system and high iron content (Fragoso et al., 2004; Volpe, 2011). OL cell body shows an increased AMPA receptor expression (Volpe, 2009), whereas OL processes extended to axons to create the myelin sheath highly express NMDA receptors (Follett et al., 2004; Salter and Fern, 2005). Overactivation of those receptors because of a massive release of glutamate, as in HI, results in OL cell death and processes retraction and then to myelination failure (Follett et al., 2004; Salter and Fern, 2005; Volpe, 2009), in accordance with our observances in the EM studies. It has been reported that TNFα has direct pro-apoptotic effects on preOL (Deng et al., 2008; Kim et al., 2011; Kaur et al., 2013). There were no differences in the expression of pro-proliferative factors as BDNF or GDNF in HI rats treated with vehicle or CBD, suggesting that CBD effects on myelination were not due to a direct pro-proliferative effect. Besides, that CBD was protecting axons or OL cells depending on the area suggests that CBD neuroprotective effects were not specific for a particular cell. Instead, it is likely that CBD’s effects on myelination after HI relied on CBD’s modulation of excitotoxicity, inflammation, and oxidative stress in the days following HI, factors which immature brain OL and premyelinating axons are particularly sensitive to (Alix, 2006; Deng et al., 2008; Kim et al., 2011; Volpe, 2011; Alix et al., 2012; Kaur et al., 2013; Back and Rosenberg, 2014; Zhao et al., 2016). However, more experiments are needed to fully determine the mechanisms of CBD’s myelination protection described in the present work.

The current standard of care for HI newborns, hypothermia, has demonstrated similar results to CBD in a similar model. In Sprague-Dawley rats undergoing an HI insult at PND7, hypothermia prevents preOL accumulation in WM 40 days after the HI insult by promoting maturation of preOL into mOL, resulting in increased MBP signal and increased number of myelinated axons (Xiong et al., 2013). Erythropoietin, another experimental neuroprotective substance administered to P7 Sprague-Dawley rats after an HI insult prevents HI-induced decrease of mOL number and MBP signal in WM and CX as assessed 14 days after the HI insult (Iwai et al., 2010). However, erythropoietin administered to P9 mice after a HI insult did not prevent HI-induced decrease of MBP signal in the long term (Fan et al., 2011). There are no reports on the effects of other promising experimental neuroprotectants, such as melatonin, in a model similar to ours. In a model of focal ischemic damage in P7 Wistar rats, melatonin prevents the subsequent decrease of mOL number and MBP signal, but that effect was assessed just 48 h after the insult (Villapol et al., 2011).

In conclusion, our study confirms that a HI insult in rats at a brain developmental stage equivalent to term infants leads to long-lasting myelination disturbance which is directly related to long-term functional disturbances. The administration of CBD single dose after the neonatal HI insult protects the maturational process of OL cells, as well as the mOL function and relationship with axons, thus, preserving normal myelination and restoring neurobehavioral function. Those results open exciting perspectives regarding a possible role for CBD in NHIE and other demyelinating pediatric conditions.

## Data Availability Statement

The datasets generated for this study are available on request to the corresponding author.

## Ethics Statement

All the animal experimental procedures were approved by the Ethical Committee for Animal Welfare of the Hospital Universitario Puerta de Hierro Majadahonda, and met the European and Spanish regulation (2010/63/EU and RD 1201/2005).

## Author Contributions

MC and FP carried out the experiments; CV and LJ-S carried out the histological studies; LG-T and MP carried out the biochemical studies; MC, LJ-S, and MP carried out the neurobehavioral tests; SA, IE, and PG carried out the electronic microscope studies; WH carried out the statistical analysis; JM-O designed the experiments; MC, WH, and JM-O wrote the manuscript.

## Funding

This work was supported by grants from the Carlos III Research Institute (ISCiii) according to the Spanish Plan for R+D+I 2008–2011 and the State Plan for Scientific and Technical Research and Innovation 2017–2019, with co-funding from the European Regional Development Funds (FEDER) (FIS-PS1600689), from the Biomedicine Program, Community of Madrid (S2010/BMD-2308) and from GW Research Ltd (GWCRI09119).

## Conflict of Interest

JM-O had a Research Agreement with GW Research Ltd (Cambridge, UK). WH was employed by GW Research Ltd. The authors declare that this study received funding from GW Research Ltd (Cambridge, UK). The funder had the following involvement with the study: decision to publish and preparation of the manuscript.
